# Supplementary material for: A complex of the lipid transport ER proteins TMEM24 and C2CD2 with band 4.1 at cell-cell contacts
Source: bioRxiv. 2023 Dec 6:2023.12.06.570396. Preprint. [Version 1] doi: 10.1101/2023.12.06.570396 (PMC10723409; doi:10.1101/2023.12.06.570396)
Supplement: 1 [file NIHPP2023.12.06.570396v1-supplement-1.pdf]

bioRxiv preprint doi: https://doi.org/10.1101/2023.12.06.570396; this version posted December 6, 2023. The copyright holder for this preprint (which was not certified by peer review) is the author/funder, who has granted bioRxiv a license to display the preprint in perpetuity. It is made available under aCC-BY 4.0 International license.

SUPPLEMENTAL FIGURE 1

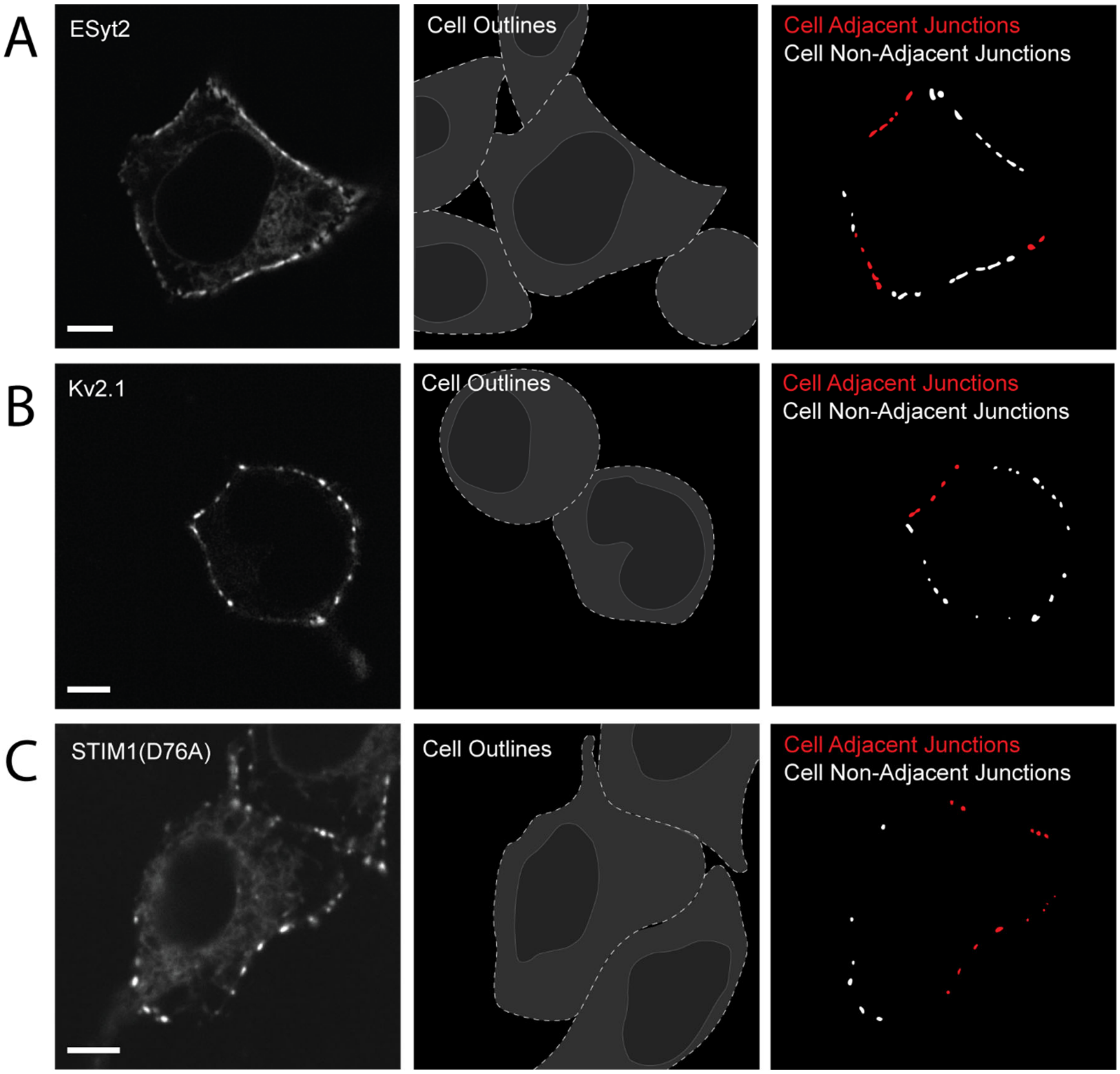

bioRxiv preprint doi: https://doi.org/10.1101/2023.12.06.570396; this version posted December 6, 2023. The copyright holder for this preprint (which was not certified by peer review) is the author/funder, who has granted bioRxiv a license to display the preprint in perpetuity. It is made available under aCC-BY 4.0 International license.

Supplemental Figure 1. Several ER/PM junction tethers tested other than TMEM24 and C2CD2 display no preference for cell-adjacent ER/PM junctions. ER/PM junctions positive for exogenously expressed E-Syt2-eGFP **(A)**, Kv2.1-eGFP **(B)** or YFP-STIM1(D76A)**(C)** in HEK293 cells show no differences in size depending on cell adjacency. Quantification of this data can be found in Figure 1B of the main text. Scale bars = 5 µm.

bioRxiv preprint doi: https://doi.org/10.1101/2023.12.06.570396; this version posted December 6, 2023. The copyright holder for this preprint (which was not certified by peer review) is the author/funder, who has granted bioRxiv a license to display the preprint in perpetuity. It is made available under aCC-BY 4.0 International license.

SUPPLEMENTAL FIGURE 2

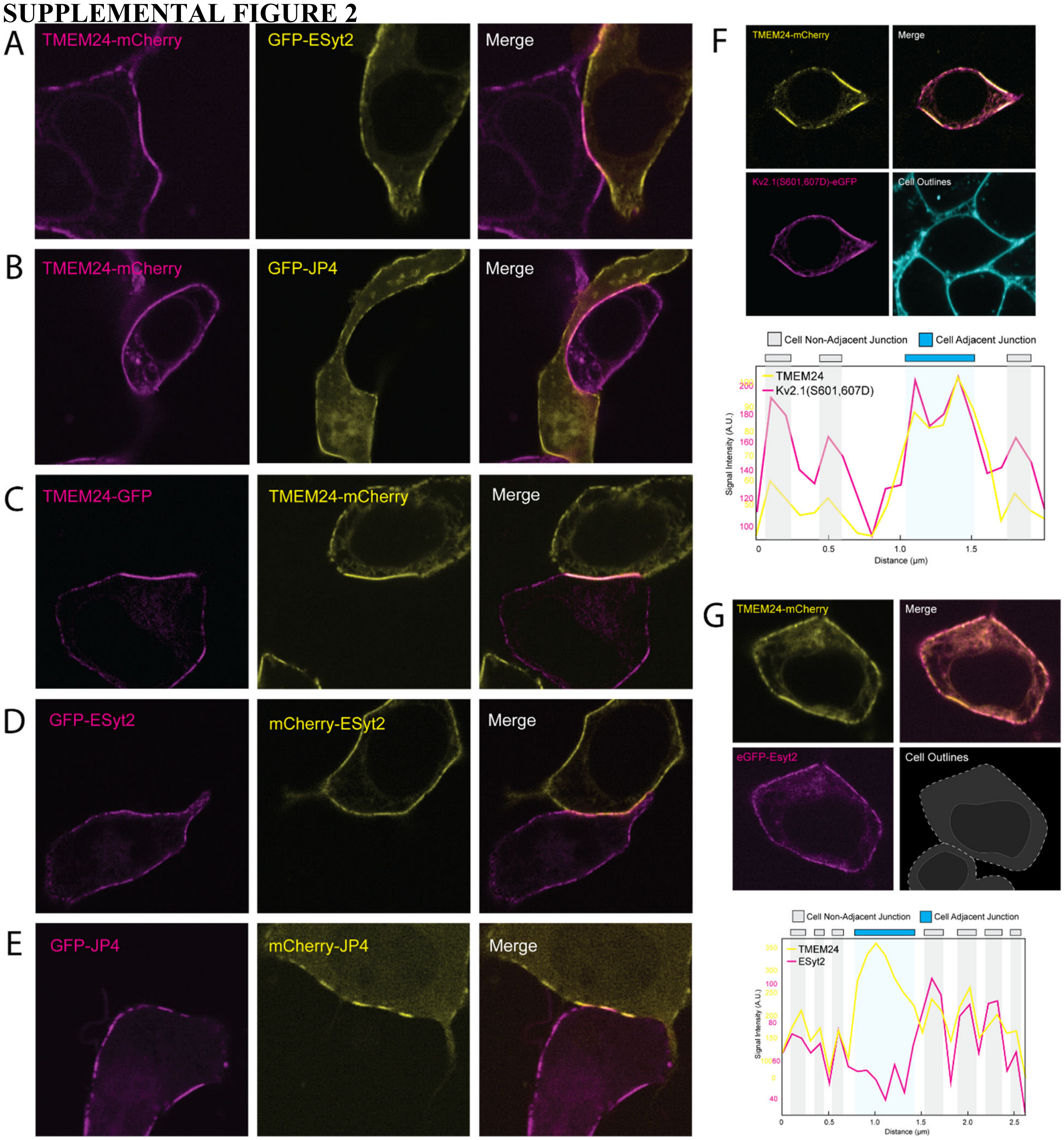

Supplemental Figure 2. ER/PM Junction Tether Behavior at the Cell-Cell Interface. HEK293 cells. TMEM24 expressed in one cell generates an enlarged ER/PM junction that is not mirrored by an EGFP-ESyt2-positive **(A)** or an JPH4-(EGFP)-positive **(B)** junction in an adjacent cell. **(C)** The accumulation of TMEM24-mCherry expressed in one cell at a cell-adjacent ER/PM junction is mirrored by the accumulation of TMEM24-GFP expressed in an adjacent cell (see also Figure 1C). **(D)** ER/PM junctions induced by mCherry-ESyt2 and GFP-ESyt2-expressed in two adjacent cells respectively, do not mirror one another across the cell-cell interface. **(E)** mCherry-JPH4 and GFP-JPH4 do not robustly mirror one another across the cell-cell interface although junctions

bioRxiv preprint doi: https://doi.org/10.1101/2023.12.06.570396; this version posted December 6, 2023. The copyright holder for this preprint (which was not certified by peer review) is the author/funder, who has granted bioRxiv a license to display the preprint in perpetuity. It is made available under aCC-BY 4.0 International license.

could be found that seemed to be symmetrically opposed. **(F** and **G)** A Kv2.1-GFP mutant constitutively bound to VAP **(F)**, but not EGFP-E-Syt2 **(G)**, colocalizes with TMEM24 at cell-adjacent ER/PM junctions. At non cell-adjacent junctions, TMEM24 colocalizes with both proteins. Quantification of line scans drawn around the cell periphery are shown below the fluorescence images.

bioRxiv preprint doi: https://doi.org/10.1101/2023.12.06.570396; this version posted December 6, 2023. The copyright holder for this preprint (which was not certified by peer review) is the author/funder, who has granted bioRxiv a license to display the preprint in perpetuity. It is made available under aCC-BY 4.0 International license.

SUPPLEMENTAL FIGURE 3

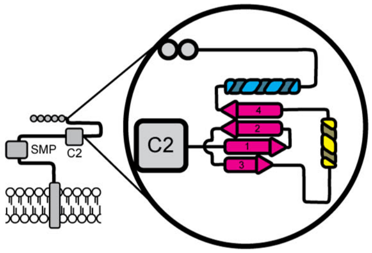


```
                               Strand 1               Strand 2
TMEM24  Human      PRPSITPTKKIELDRTIMPDGTIVTTVTTVQSR      455
        Mouse      PRPSITPTKKIELDRTIMPDGTVVTTVTTVQSR      455
        Zebrafish  LRSSVTPSKKVDVDRTIMPDGTIVTTVTTIQSR      460
        Xenopus    SRTNITPTKKVEMDRTVMPDGTIVTTVTTIQSR      418
C2CD2   Human      PIPPPVPAAKIEKDRTVMPCGTVVTTVTAVKTK      426
        Mouse      PAPPPVSAAKIEKDRTVMPCGTVVTTVTAVKTK      421
        Zebrafish  QTPTPAPTKRVEMDRTVMPCGTVVTTVTAVRSK      416
        Xenopus    TFPTPVAAKKVEKDRTVMPCGTVVTTVTSVTSK      407
                        . : ::: ***:** **:*****:: ::

                                        Strand 3
TMEM24  Human      PRIDGK-----L-DSPSRSPSKVEVTEKTTTVLSESSGPSNTSH---SSSRDSHLSNGLD      506
        Mouse      PRVDGK-----L-DSPSRSPSKVEVTEKMTTVLSESSGPSNASH---SSSRESHLSNGLD      506
        Zebrafish  LKLDRK-----LGESPSRSPSKVEVTEKPPTVLTNSLQSTSGSG----TPSKGTLPNGLD      511
        Xenopus    LRLDGK-----I-DSPSRSPSKVEVTENKPIFMPRSCSPGSS-P---SGSGGSPVSLRLD      468
C2CD2   Human      PRVDVGRASPLSSDSPVKTPIKVKVIEKDISVQAIACRSAPVSKTLSSSDTELLVLNGSD      486
        Mouse      PRFDTGRATPLNSESPVRTPVTVKVIEKDISVQAISCHSAPVSKTFSSSDTELLVLNGSD      481
        Zebrafish  PGR----PLPPESKS----PSKNKLAERRVSEQP-SILGAKVSKALSSSDTELLILNGTD      467
        Xenopus    PRLDG-KSPVQSYESPAKTPPKVKVIERDFSVQTMPSESMVVSKALSSSDTELLFLNGSD      466
                                .*    * . :: *.                          .    *

                   Alpha Helix 1                  Strand 4    Alpha Helix 2
TMEM24  Human      PVAETAIRQLTEPSGRVAKKTPTKRSTLIISGVSKVPIAQDE-LALSLGYAASLEASVQD      565
        Mouse      PVAETAIRQLTEPSGRAAKKTPTKRSTLIISGVSKVPIAQDE-LALSLGYAASLEASMQD      565
        Zebrafish  PVAETAIRQLTESASKVPKKTPTKRSTLIISGVSKVPITEDD-SALSVGYAAAMDAALQG      570
        Xenopus    PVAETAIRQLTEPGNKPAKKTPTKRSTLIISGVSKVPIGQDE-MALCLGYAASMDASLQG      527
C2CD2   Human      PVAEVAIRQLSESS-KLKLKSPRKKSTIIISGISKTSLSQDHDAALMQGYTASVDSTHQE      545
        Mouse      PVAEVAIRQLSESS-KLKLKSPRKKSTIIISGISKTSLSQDHNAALMLDYAASMDSTNQG      540
        Zebrafish  PVAEAAIRQLHESA-KQKLKSPVKKSTIIISGVSKAPLSQDEEMSLMAGYAAAMDASMSG      526
        Xenopus    PVAEAAIRQLRESS-KQSFKSPRKKSTIIISGISKTSVSQDEEVSLMLNYAEAMDNSVMN      525
                   ****.***** * . :   *:* *:**:****:**.: :*.  :*  .*: ::: :
```

Supplemental Figure 3. Sequence alignments of portions of the TMEM24 and C2CD2 C-termini across species demonstrating conservation of the α-helixes and β-strands. The cartoon at top left shows a schematic view of a TMEM24 monomer, with an enlarged view of the Alphafold predicted structural motifs (colored) within its 414-630 amino acid region.

bioRxiv preprint doi: https://doi.org/10.1101/2023.12.06.570396; this version posted December 6, 2023. The copyright holder for this preprint (which was not certified by peer review) is the author/funder, who has granted bioRxiv a license to display the preprint in perpetuity. It is made available under aCC-BY 4.0 International license.

SUPPLEMENTAL FIGURE 4

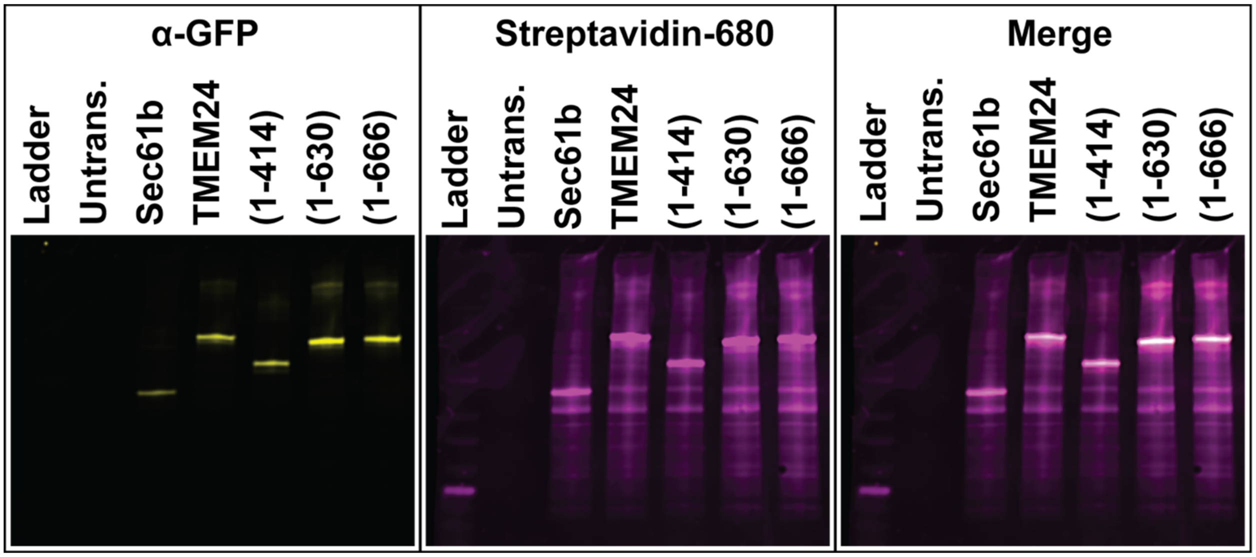

Supplemental Figure 4. Streptavidin affinity-purification of cell extracts expressing the constructs indicated and homogenized after the APEX2 reaction. Anti-GFP Western blots and streptavidin overlay of material affinity-purified on streptavidin bead. Numbers in parenthesis indicate amino acid boundaries of TMEM24 fragments used. Note the high degree of self-biotinylation for each construct.

bioRxiv preprint doi: https://doi.org/10.1101/2023.12.06.570396; this version posted December 6, 2023. The copyright holder for this preprint (which was not certified by peer review) is the author/funder, who has granted bioRxiv a license to display the preprint in perpetuity. It is made available under aCC-BY 4.0 International license.

SUPPLEMENTAL FIGURE 5

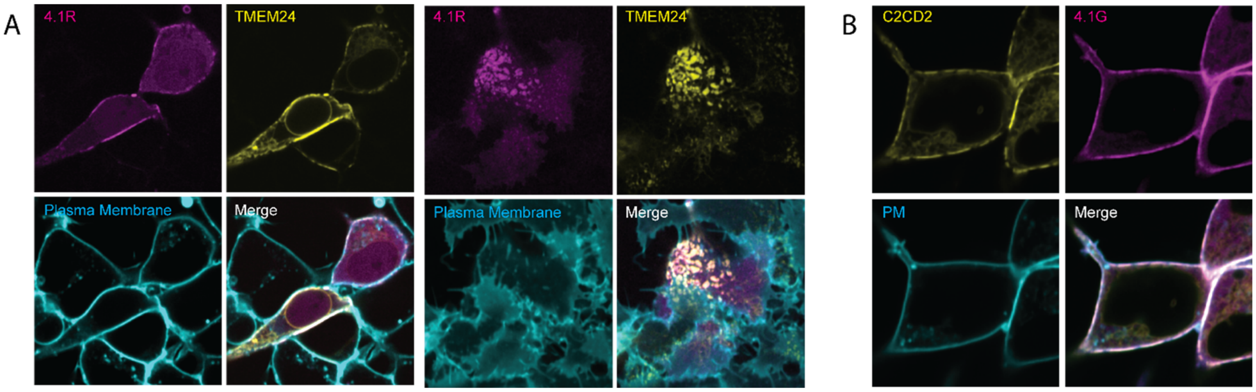

Supplemental Figure 5. The TMEM24-Band 4.1 partnership is observed also with their paralogues. (A) Colocalization of TMEM24-mCherry with band 4.1R-GFP at ER/PM junctions of HEK293 cells as seen in both a mid-cell confocal z-slice and at the basal surface. **(B)** Colocalization of C2CD2-eGFP with mCherry-4.1G.

bioRxiv preprint doi: https://doi.org/10.1101/2023.12.06.570396; this version posted December 6, 2023. The copyright holder for this preprint (which was not certified by peer review) is the author/funder, who has granted bioRxiv a license to display the preprint in perpetuity. It is made available under aCC-BY 4.0 International license.

Supplemental Table 1. Proteins identified in the APEX2 screen. Proteins are ranked by significance value (student's t-test), comparing grouped mass spec hits of protein constructs that inhabit ER/PM junctions (full length TMEM24, TMEM24(1-630) and TMEM24(1-666)) versus constructs that do not inhabit ER/PM junctions (untransfected, sec61β, and TMEM24(1-414)). Significant hits are highlighted in cyan, band 4.1 family members are highlighted in yellow, and MPP family members are highlighted in magenta. The full list of all proteins identified with 2+ peptides is included in the second tab.
